# Species conservation profiles of a random sample of world spiders II: Gnaphosidae to Nemesiidae

**DOI:** 10.3897/BDJ.6.e26203

**Published:** 2018-06-29

**Authors:** Sini Seppälä, Sérgio Henriques, Michael L Draney, Stefan Foord, Alastair T Gibbons, Luz A Gomez, Sarah Kariko, Jagoba Malumbres-Olarte, Marc Milne, Cor J Vink, Pedro Cardoso

**Affiliations:** 1 Finnish Museum of Natural History, University of Helsinki, Helsinki, Finland; 2 IUCN SSC Spider & Scorpion Specialist Group, Helsinki, Finland; 3 University College London, London, United Kingdom; 4 University of Wisconsin-Green Bay, Green Bay, United States of America; 5 University of Venda, Thohoyandou, South Africa; 6 University of Nottingham, Nottingham, United Kingdom; 7 Universidad Nacional de Colombia, Bogotá, Colombia; 8 Museum of Comparative Zoology, Harvard University, Cambridge, United States of America; 9 University of Copenhagen, Copenhagen, Denmark; 10 University of Barcelona, Barcelona, Spain; 11 University of Indianapolis, Indianapolis, United States of America; 12 Canterbury Museum, Christchurch, New Zealand

**Keywords:** Araneae, Arthropoda, conservation, endangered species, extinction risk, geographical range, IUCN.

## Abstract

**Background:**

The IUCN Red List of Threatened Species is the most widely used information source on the extinction risk of species. One of the uses of the Red List is to evaluate and monitor the state of biodiversity and a possible approach for this purpose is the Red List Index (RLI). For many taxa, mainly hyperdiverse groups, it is not possible within available resources to assess all known species. In such cases, a random sample of species might be selected for assessment and the results derived from it extrapolated for the entire group - the Sampled Red List Index (SRLI). The current contribution is the second in four papers that will constitute the baseline of a future spider SRLI encompassing 200 species distributed across the world.

**New information:**

A sample of 200 species of spiders were randomly selected from the World Spider Catalogue, an updated global database containing all recognised species names for the group. The 200 selected species where divided taxonomically at the family level and the familes were ordered alphabetically. In this publication, we present the conservation profiles of 45 species belonging to the families alphabetically arranged between Gnaphosidae and Nemesiidae, which encompassed Gnaphosidae, Idiopidae, Linyphiidae, Liocranidae, Lycosidae, Micropholcommatidae, Mysmenidae and Nemesiidae.

## Introduction

The IUCN Red List of Threatened Species is the most widely used information source on the extinction risk of species (Lamoreux et al. 2003, Rodrigues et al. 2006, Mace et al. 2008, see also Cardoso et al. 2011, Cardoso et al. 2012). It is based on a number of objective criteria, which are relatively easy to apply when adequate information is available (IUCN 2001). The Red List has been used to raise awareness about threatened species, guide conservation efforts and funding, set priorities for protection, measure site irreplaceability and vulnerability and influence environmental policies and legislation (Gardenfors et al. 2001, Rodrigues et al. 2006, Mace et al. 2008, Martín-López et al. 2009).

One of the uses of the Red List is to evaluate and monitor the state of biodiversity and a possible approach for this purpose is the Red List Index (RLI). The RLI helps to develop a better understanding of which taxa, regions or ecosystems are declining or improving their conservation status. It provides policy-makers, stakeholders, conservation practitioners and the general public with sound knowledge of biodiversity status and change and tools with which to make informed decisions. The RLI uses weight scores based on the Red List status of each of the assessed species. These scores range from 0 (Least Concern) to 5 (Extinct/Extinct in the Wild). Summing these scores across all species, relating them to the worst-case scenario - all species extinct and comparing two or more points in time, gives us an indication of how biodiversity is doing. At a global level, the RLI has been calculated for birds (Butchart et al. 2004, Hoffmann et al. 2010), mammals (Hoffmann et al. 2011), amphibians (Hoffmann et al. 2010), corals (Butchart et al. 2010) and cycads (United Nations 2015).

For many taxa, mainly hyperdiverse groups, it is not possible within available resources to assess all known species. In such cases, a random sample of species might be selected for assessment and the results derived from it extrapolated for the entire group - the Sampled Red List Index (SRLI, Baillie et al. 2008). The SRLI is now being developed for plants (Brummitt et al. 2015) and efforts towards a SRLI of butterflies (Lewis and Senior 2010) and Odonata are also in progress (Clausnitzer et al. 2009).

Spiders currently comprise over 47000 species described at a global level (World Spider Catalog 2017). Of these, only 199 species (0.4%) have beed assessed (www.redlist.org), of which the vast majority are from the Seychelles Islands or belong to the golden-orb weavers, Nephilidae (e.g. Kuntner et al. 2017). To these, a large number will be added in the near future, such as 55 species endemic to the Madeira and Selvagens archipelagos and 25 endemic to the Azores, all in Portugal (Cardoso et al. 2017, Borges et al. submitted). The vast majority of spiders assessed to date are therefore either regionally or taxonomically clustered and do not represent the group as a whole. The current contribution is the second in four papers (Seppälä et al. 2018) that will constitute the baseline of a future spider SRLI encompassing 200 species distributed across the world.

## Methods

A sample of 200 species of spiders were randomly selected from the World Spider Catalogue (2018), an updated global database containing all recognised species names for the group. The 200 selected species where divided taxonomically to the family level, and those familes were ordered alphabetically. In this publication, we present the conservation profiles of 45 species belonging to the families alphabetically arranged between Gnaphosidae and Nemesiidae, which encompassed Gnaphosidae, Idiopidae, Linyphiidae, Liocranidae, Lycosidae, Micropholcommatidae, Mysmenidae and Nemesiidae.

Species data were collected from all taxonomic bibliography available at the World Spider Catalogue (2018), complemented by data in other publications found through Google Scholar and georeferrenced points made available through the Global Biodiversity Information Facility (www.gbif.org) and also other sources (https://www.biodiversitylibrary.org; https://login.webofknowledge.com; http://srs.britishspiders.org.uk; http://symbiota4.acis.ufl.edu/scan/portal; https://lepus.unine.ch; http://www.tuite.nl/iwg/Araneae/SpiBenelux/?species; https://atlas.arages.de; https://arachnology.cz/rad/araneae-1.html; http://www.ennor.org/iberia/). Whenever possible, with each species record, we also collected additional information, namely habitat type and spatial error of coordinates.

For all analyses, we used the R package red - IUCN red-listing tools (Cardoso 2017). This package performs a number of spatial analyses based on either observed occurrences or estimated ranges. Functions include calculating Extent of Occurrence (EOO), Area of Occupancy (AOO), mapping species ranges, species distribution modelling using climate and land cover, calculating the Red List Index for groups of species, amongst others. In this work, the EOO and AOO were calculated in one of two ways:

- for extremely range-restricted species for which we assumed knowledge of the full range, these values were classified as observed, the minimum convex polygon encompassing all observations used to calculate the EOO and the 2 km x 2 km cells known to be occupied used to calculate the AOO. When the EOO was smaller than the AOO, it was made equal as per the IUCN guidelines (IUCN Standards and Petitions Subcommittee 2017).

- for widespread species or those for which we did not have confidence to know the full range, we performed species distribution modelling (SDM). This was done based on both climatic (Fick and Hijmans 2017) and landcover (Tuanmu and Jetz 2014) datasets, at an approximately 1x1 km resolution. Before modelling, the world layers were cropped to the region of interest for each species and reduced to four layers through a PCA to avoid overfitting. In addition, latitude and longitude were used as two extra layers to avoid the models predicting presences much beyond the known region following the precautionary principle. We then used the Maxent method (Phillips et al. 2006) implemented in the R package red. Isolated patches outside the original distribution polygon were excluded from maps to avoid overestimation of EOO and AOO values. All final maps and values were checked and validated by our own expert opinion. KMLs derived from these maps were also produced using the red package. The cells (2x2 km), predicted to be occupied, were used to calculate the AOO. When the EOO was smaller than the AOO, it was made equal as per the IUCN guidelines (IUCN Standards and Petitions Subcommittee 2017).

To infer possible changes in range and/or abundance and for forest species only, we have also consulted the Global Forest Watch portal (World Resources Institute 2014), looking for changes in forest cover during the last 10 years that could have affected the species.

## Supplementary Material

Supplementary material 1 Distribution of *Berlandina kolosvaryi* Caporiacco, 1947 Data type: Distribution
File: oo_174617.kml Cardoso, P.

Supplementary material 2 Distribution of *Drassyllus excavatus* (Schenkel, 1963) Data type: Distribution
File: oo_170265.kml Cardoso, P.

Supplementary material 3 Distribution of *Gnaphosa kankhalae* Biswas & Roy, 2008 Data type: Distribution
File: oo_170269.kml Cardoso, P.

Supplementary material 4 Distribution of *Gnaphosa tenebrosa* Fox, 1938 Data type: Distribution
File: oo_170270.kml Cardoso, P.

Supplementary material 5 Distribution of *Leptodrassus croaticus* Dalmas, 1919 Data type: Distribution
File: oo_170271.kml Cardoso, P.

Supplementary material 6 Distribution of *Orodrassus coloradensis* (Emerton, 1877) Data type: Distribution
File: oo_195709.kml Cardoso, P.

Supplementary material 7 Distribution of *Scotophaeus nigrosegmentatus* (Simon, 1895) Data type: Distribution
File: oo_170273.kml Cardoso, P.

Supplementary material 8 Distribution of *Urozelotes mysticus* Platnick & Murphy, 1984 Data type: Distribution
File: oo_195708.kml Cardoso, P.

Supplementary material 9 Distribution of *Zelotes anthereus* Chamberlin, 1936 Data type: Distribution
File: oo_170275.kml Cardoso, P.

Supplementary material 10 Distribution of *Zelotes ashae* Tikader & Gajbe, 1976 Data type: Distribution
File: oo_170276.kml Cardoso, P.

Supplementary material 11 Distribution of *Zelotes mulanjensis* FitzPatrick, 2007 Data type: Distribution
File: oo_170279.kml Cardoso, P.

Supplementary material 12 Distribution of *Cantuaria wanganuiensis* (Todd, 1945) Data type: Distribution
File: oo_170280.kml Cardoso, P.

Supplementary material 13 Distribution of *Cataxia bolganupensis* (Main, 1985) Data type: Distribution
File: oo_170281.kml Cardoso, P.

Supplementary material 14 Distribution of *Galeosoma robertsi* Hewitt, 1916 Data type: Distribution
File: oo_170282.kml Cardoso, P.

Supplementary material 15 Distribution of *Agyneta flibuscrocus* Dupérré, 2013 Data type: Distribution
File: oo_199077.kml Cardoso, P.

Supplementary material 16 Distribution of *Agyneta mongolica* (Loksa, 1965) Data type: Distribution
File: oo_170284.kml Cardoso, P.

Supplementary material 17 Distribution of *Ceratinella brunnea* Emerton, 1882 Data type: Distribution
File: oo_170285.kml Cardoso, P.

Supplementary material 18 Distribution of *Mansuphantes ovalis* (Tanasevitch, 1987) Data type: Distribution
File: oo_170286.kml Cardoso, P.

Supplementary material 19 Distribution of *Parafroneta marrineri* (Hogg, 1909) Data type: Distribution
File: oo_170287.kml Cardoso, P.

Supplementary material 20 Distribution of *Pelecopsis alticola* (Berland, 1936) Data type: Distribution
File: oo_212566.kml Cardoso, P.

Supplementary material 21 Distribution of *Pelecopsis parallela* (Wider, 1834) Data type: Distribution
File: oo_212565.kml Cardoso, P.

Supplementary material 22 Distribution of *Scotinotylus sacer* (Crosby, 1929) Data type: Distribution
File: oo_170291.kml Cardoso, P.

Supplementary material 23 Distribution of *Tapinocyba suganamii* Saito & Ono, 2001 Data type: Distribution
File: oo_170292.kml Cardoso, P.

Supplementary material 24 Distribution of *Tiso aestivus* (L. Koch, 1872) Data type: Distribution
File: oo_170293.kml Cardoso, P.

Supplementary material 25 Distribution of *Troxochrus triangularis* Tanasevitch, 2013 Data type: Distribution
File: oo_195707.kml Cardoso, P.

Supplementary material 26 Distribution of *Arabelia pheidoleicomes* Bosselaers, 2009 Data type: Distribution
File: oo_170295.kml Cardoso, P.

Supplementary material 27 Distribution of *Neoanagraphis pearcei* Gertsch, 1941 Data type: Distribution
File: oo_170296.kml Cardoso, P.

Supplementary material 28 Distribution of *Arctosa villica* (Lucas, 1846) Data type: Distribution
File: oo_199025.kml Cardoso, P.

Supplementary material 29 Distribution of *Hippasa greenalliae* (Blackwall, 1867) Data type: Distribution
File: oo_170298.kml Cardoso, P.

Supplementary material 30 Distribution of *Hogna exsiccatella* (Strand, 1916) Data type: Distribution
File: oo_170299.kml Cardoso, P.

Supplementary material 31 Distribution of *Hogna kankunda* Roewer, 1959 Data type: Distribution
File: oo_170300.kml Cardoso, P.

Supplementary material 32 Distribution of *Lycosa fuscana* Pocock, 1901 Data type: Distribution
File: oo_170301.kml Cardoso, P.

Supplementary material 33 Distribution of *Lycosa goliathus* Pocock, 1901 Data type: Distribution
File: oo_170302.kml Cardoso, P.

Supplementary material 34 Distribution of *Lycosa iranii* Pococks, 1901 Data type: Distribution
File: oo_170303.kml Cardoso, P.

Supplementary material 35 Distribution of *Pardosa izabella* Chamberlin & Ivie, 1942 Data type: Distribution
File: oo_170304.kml Cardoso, P.

Supplementary material 36 Distribution of *Pardosa kupupa* (Tikader, 1970) Data type: Distribution
File: oo_195706.kml Cardoso, P.

Supplementary material 37 Distribution of *Pardosa novitatis* (Strand, 1916) Data type: Distribution
File: oo_170306.kml Cardoso, P.

Supplementary material 38 Distribution of *Pardosa pusiola* (Thorell, 1891) Data type: Distribution
File: oo_199024.kml Cardoso, P.

Supplementary material 39 Distributon of *Pardosa sinensis* in, Wang, Peng & Xie, 1995 Data type: Distribution
File: oo_212567.kml Cardoso, P.

Supplementary material 40 Distribution of *Schizocosa avida* (Walckenaer, 1837) Data type: Distribution
File: oo_170310.kml Cardoso, P.

Supplementary material 41 Distribution of *Trochosa longa* Qu, Peng & Yin, 2010 Data type: Distribution
File: oo_170311.kml Cardoso, P.

Supplementary material 42 Distribution of *Zenonina fusca* Caporiacco, 1941 Data type: Distribution
File: oo_170312.kml Cardoso, P.

Supplementary material 43 Distribution of *Pua novaezealandiae* Forster, 1959 Data type: Distribution
File: oo_170313.kml Cardoso, P.

Supplementary material 44 Distribution of *Mosu huogou* Miller, Griswold & Yin, 2009 Data type: Distribution
File: oo_170314.kml Cardoso, P.

Supplementary material 45 Distribution of *Calisoga sacra* Chamberlin, 1937 Data type: Distribution
File: oo_170315.kml Cardoso, P.

## References

[B3805993] Agnarsson I. (1996). Íslenskar köngulaer. Fjölrit Náttúrufraeðistofnunar, 31, 1 – 175. Audouin, V. (1826) Explication sommaire des planches d'arachnides de l'Egypte et de la Syrie. Histoire Naturelle.

[B3808461] Agnew Charles W., Dean David A., Smith J. W. (1985). Spiders collected from peanuts and non-agricultural habitats in the Texas West Cross-Timbers. The Southwestern Naturalist.

[B3787590] Allred Dorald M., Gertsch Willis J. (1975). Spiders and Scorpions from Northern Arizona and Southern Utah. Journal of Arachnology.

[B4035825] Baillie Jonathan E. M., Collen Ben, Amin Rajan, Akcakaya H. Resit, Butchart Stuart H. M., Brummitt Neil, Meagher Thomas R., Ram Mala, Hilton-Taylor Craig, Mace Georgina M. (2008). Toward monitoring global biodiversity. Conservation Letters.

[B3804246] Banks Nathan (1901). Some Arachnida from New Mexico. Proceedings of the Academy of Natural Sciences of Philadelphia.

[B3806456] Barrientos J. A. (1979). La colección de Araneídos del Departamento de Zoolo-gía de la Universidad de Salamanca, II: familias Lycosidae, Oxyopidae y Pisauridae (Araneae). Boletín de la Asociación Española de Entomología.

[B3788328] Becker L. (1896). Les arachnides de Belgique. Annales du Musée Royal d'Histoire Naturelle de Belgique.

[B3805542] Berland L. (1931). Araignées des Iles Auckland et Campbell. Records of the Canterbury Museum.

[B3756413] Biswas B., Roy R. (2008). Description of six new species of spiders of the genera *Lathys* (Family: Dictynidae), *Marpissa* (Family: Salticidae), *Misumenoides* (Family: Thomisidae), *Agroeca* (Family: Clubionidae), *Gnaphosa* (Family: Gnaphosidae) and *Flanona* (Family: Lycosidae) from India. Zenodo.

[B3808411] Biswas V., Raychaudhuri D. (2003). Wolf spiders of Bangladesh: genus *Pardosa* C. L. Koch (Araneae: Lycosidae). Records of the Zoological Survey of India.

[B3806543] Biswas V., Raychaudhuri D. (2007). New record of wolf spiders (Araneae: Lycosidae) of the genus *Hippasa* Simon from Bangladesh. Journal of the Bombay Natural History Society.

[B3808481] Blackwall John (1846). VII. Notice of spiders captured by Professor Potterin Canada, with descriptions of such species as appear to be new to science. Journal of Natural History.

[B3788377] Blackwall J. (1864). A history of the spiders of Great Britain and Ireland.

[B3806678] Blackwall J. (1867). Descriptions of several species of East Indian spiders, apparently to be new or little known to arachnologists. Annals and Magazine of Natural History.

[B3805508] Blest A. D. (1979). The spiders of New Zealand. Part V. Linyphiidae-Mynoglenidae. Otago Museum Bulletin.

[B3788318] Bosenberg W. (1902). Die Spinnen Deutschlands. II-IV.. Zoologica (Stuttgart).

[B3806405] Bosmans Robert (2011). On some new or rare spider species from Lesbos, Greece (Araneae: Agelenidae, Amaurobiidae, Corinnidae, Gnaphosidae, Liocranidae). Arachnologische Mitteilungen.

[B3806372] Bosselaers J (2009). Studies in Liocranidae (Araneae): redescriptions and transfers in *Apostenus* Westring and *Brachyanillus* Simon, as well as description of a new genus.. Zootaxa.

[B3806003] Braendegaard J. (1946). The spiders (Araneina) of East Greenland: a faunistic and zoogeographical investigation. Meddelelser om Grønland.

[B3808452] Breene R. G., Dean D. A., Nyffeler M., Edwards G. B. (1993). Biology, Predation Ecology, and Significance of Spiders in Texas Cotton Ecosystems with a Key to Species.

[B3775781] Breitling R., Bauer T., Schäfer M., Morano E., Barrientos J. A., Blick T. (2016). Phantom spiders 2: More notes on dubious spider species from Europe. Arachnologische Mitteilungen/Arachnology Letters.

[B4035841] Brummitt Neil A, Bachman Steven P, Griffiths-Lee Janine, Lutz Maiko, Moat Justin F, Farjon Aljos, Donaldson John S, Hilton-Taylor Craig, Meagher Thomas R, Albuquerque Sara, Aletrari Elina, Andrews A Kei, Atchison Guy, Baloch Elisabeth, Barlozzini Barbara, Brunazzi Alice, Carretero Julia, Celesti Marco, Chadburn Helen, Cianfoni Eduardo, Cockel Chris, Coldwell Vanessa, Concetti Benedetta, Contu Sara, Crook Vicki, Dyson Philippa, Gardiner Lauren, Ghanim Nadia, Greene Hannah, Groom Alice, Harker Ruth, Hopkins Della, Khela Sonia, Lakeman-Fraser Poppy, Lindon Heather, Lockwood Helen, Loftus Christine, Lombrici Debora, Lopez-Poveda Lucia, Lyon James, Malcolm-Tompkins Patricia, McGregor Kirsty, Moreno Laura, Murray Linda, Nazar Keara, Power Emily, Quiton Tuijtelaars Mireya, Salter Ruth, Segrott Robert, Thacker Hannah, Thomas Leighton J, Tingvoll Sarah, Watkinson Gemma, Wojtaszekova Katerina, Nic Lughadha Eimear M (2015). Green plants in the Red: A baseline global assessment for the IUCN sampled red list index for plants. PloS One.

[B4035568] Butchart Stuart H M, Stattersfield Alison J, Bennun Leon A, Shutes Sue M, Akçakaya H Resit, Baillie Jonathan E M, Stuart Simon N, Hilton-Taylor Craig, Mace Georgina M (2004). Measuring global trends in the status of biodiversity: red list indices for birds.. PLoS Biology.

[B4035699] Butchart Stuart H M, Walpole Matt, Collen Ben, van Strien Arco, Scharlemann Jörn P W, Almond Rosamunde E A, Baillie Jonathan E M, Bomhard Bastian, Brown Claire, Bruno John, Carpenter Kent E, Carr Geneviève M, Chanson Janice, Chenery Anna M, Csirke Jorge, Davidson Nick C, Dentener Frank, Foster Matt, Galli Alessandro, Galloway James N, Genovesi Piero, Gregory Richard D, Hockings Marc, Kapos Valerie, Lamarque Jean-Francois, Leverington Fiona, Loh Jonathan, McGeoch Melodie A, McRae Louise, Minasyan Anahit, Hernández Morcillo Monica, Oldfield Thomasina E E, Pauly Daniel, Quader Suhel, Revenga Carmen, Sauer John R, Skolnik Benjamin, Spear Dian, Stanwell-Smith Damon, Stuart Simon N, Symes Andy, Tierney Megan, Tyrrell Tristan D, Vié Jean-Christophe, Watson Reg (2010). Global biodiversity: indicators of recent declines.. Science.

[B3756403] Caporiacco L. (1947). Arachnida Africae Orientalis, a dominibus Kittenberger, Kovács et Bornemisza lecta, in Museo Nationali Hungarico servata. Annales Historico-Naturales Musei Nationalis Hungarici.

[B3804057] Caporiacco L. di (1934). Aracnidi dell'Himalaia e del Karakoram raccolti dalla Missione Italiana al Karakoram (1929 – VII). Memorie della Società Entomologica Italiana, Genova.

[B3910840] Caporiacco L. di (1941). Arachnida (esc. Acarina). Missione Biologica Sagan-Omo, Reale Accademia d’Italia. Roma.

[B4035506] Cardoso P., Borges P. A. V., Triantis K. A., Ferrández M. A., Martín J. L. (2011). Adapting the IUCN Red List criteria for invertebrates. Biological Conservation.

[B4035527] Cardoso Pedro, Borges Paulo A. V., Triantis Kostas A., Ferrández Miguel A., Martín José L. (2012). The underrepresentation and misrepresentation of invertebrates in the IUCN Red List. Biological Conservation.

[B4036129] Cardoso Pedro (2017). red - an R package to facilitate species red list assessments according to the IUCN criteria.. Biodiversity Data Journal.

[B4036063] Cardoso Pedro, Crespo Luís, Silva Isamberto, Borges Paulo, Boieiro Mário (2017). Species conservation profiles of endemic spiders (Araneae) from Madeira and Selvagens archipelagos, Portugal. Biodiversity Data Journal.

[B3757232] Chamberlin RV (1937). On two genera of trap-door spiders from California.. Bulletin of the University of Utah, Biological Series.

[B3772696] Chamberlin R. V. (1936). Further records and descriptions of North American Gnaphosidae. American Museum Novitates.

[B3757135] Chamberlin R. V., Ivie W. (1942). A hundred new species of American spiders. Bulletin of the University of Utah.

[B3804236] Chamberlin R. V. (1949). On some American spiders of the family Erigonidae. Annals of the Entomological Society of America.

[B3788338] Chyzer C., Kulczynski W. (1894). Araneae Hungariae. Budapest.

[B4035971] Clausnitzer V, Kalkman VJ, Ram M, Collen B, Baillie JEM, Bedjanic M, Darwall WRT, Dijkstra KDB, Dow R, Hawking J, Karube H, Malikova E, Paulson D, Schutte K, Suhling F, Villanueva RJ, von Ellenrieder N, Wilson K (2009). Odonata enter the biodiversity crisis debate: The first global assessment of an insect group.. Biological Conservation.

[B3804226] Crosby C. R., Bishop S. C. (1925). Studies in New York spiders; genera: *Ceratinella* and *Ceraticelus*. New York State Museum Bulletin.

[B3805722] Crosby C. R. (1929). Studies in North American spiders: the genus *Cochlembolus* (Araneina). Entomological News.

[B3788348] Dahl F. (1886). Monographie der Erigone-Arten im Thorell' schen. nebst anderen Beiträgen zur Spinnenfauna Schleswig Holsteins.. Schriften des Naturwissenschaftlichen Vereins für Schleswig-Holstein.

[B3756775] Dalmas Raymond de (1919). Catalogue des Araignées du genre *Leptodrassus* (Gnaphosidé) d'après les matériaux de la collection E. Simon au Muséum d'Histoire Naturelle. Bulletin du Muséum National d'Histoire Naturelle..

[B3806713] Dey A., Debnath S., Dennarma B., Chaudhuri P. S. (2013). A preliminary study on spider from a household garden (artificial mixed plantation) in West Tripura, India. Journal of Research in biology.

[B3808380] Dhali D. C., Saha S., Raychaudhuri D. (2017). Litter and ground dwelling spiders (Araneae: Arachnida) of reserve forests of Dooars, West Bengal. World Scientific News.

[B3909572] Dippenaar-Schoeman A. S., Jocqué R. (1997). African Spiders: An Identification manual.

[B3809336] Dippenaar S. M., Dippenaar-Schoeman A. S., Modiba M. A., Khoza T. T. (2008). A checklist of the spiders (Arachnida, Araneae) of the Polokwane Nature Reserve, Limpopo Province, South Africa. Koedoe.

[B3756887] Dupérré Nadine (2013). Taxonomic revision of the spider genera *Agyneta* and *Tennesseellum* (Araneae, Linyphiidae) of North America north of Mexico with a study of the embolic division within Micronetinae sensu Saaristo & Tanasevitch 1996. Zootaxa.

[B3808442] Durán-Barrón C. G., Francke O. F., Pérez-Ortiz T. M. (2009). Diversidad de arañas (Arachnida: Araneae) asociadas con viviendas de la ciudad de México (Zona Metropolitana). Revista Mexicana de Biodiversidad.

[B3805732] Eskov K. Y., Marusik Y. M. (1994). New data on the taxonomy and faunistics of North Asian linyphiid spiders (Aranei
Linyphiidae). Arthropoda Selecta.

[B3805643] Fage L., Simon E. (1936). Arachnida. III. Pedipalpi, Scorpiones, Solifuga et Araneae (1re partie). Mission Scientifique de l'Omo.

[B4036148] Fick Stephen E., Hijmans Robert J. (2017). WorldClim 2: new 1-km spatial resolution climate surfaces for global land areas. International Journal of Climatology.

[B3804078] FitzPatrick M. J. (2007). A taxonomic revision of the Afrotropical species of *Zelotes* (Arachnida: Araneae: Gnaphosidae). Bulletin of the British Arachnological Society.

[B3809440] Forster R. R. (1959). The spiders of the family Symphytognathidae. Transactions and Proceedings of the Royal Society of New Zealand.

[B3756848] Forster R. R. (1968). The spiders of New Zealand. Part II. Ctenizidae, Dipluridae.. Otago Museum Bulletin.

[B3756732] Fox I. (1938). Notes on North American spiders of the families Gnaphosidae, Anyphaenidae and Clubionidae. Iowa State College Journal of Science.

[B3806563] Gajbe Pawan (2003). Checklist of spiders (Arachnida: Araneae) of Madhya Pradesh and Chhattisgarh. Zoos' Print Journal.

[B4035538] Gardenfors Ulf, Hilton-Taylor Craig, Mace Georgina M., Rodriguez Jon Paul (2001). The application of IUCN Red List Criteria at Regional Levels. Conservation Biology.

[B3920515] GBIF.org GBIF Occurrence Download. https://doi.org/10.15468/dl.extw7o.

[B3787571] GBIF.org GBIF Occurrence Download. https://doi.org/10.15468/dl.pzjndp.

[B3805682] GBIF.org GBIF Occurrence Download. https://doi.org/10.15468/dl.9opwom.

[B3804137] GBIF.org GBIF Occurrence Download. https://doi.org/10.15468/dl.mja0fa.

[B3804047] GBIF.org GBIF Occurrence Download. https://doi.org/10.15468/dl.eismdr.

[B3808431] GBIF.org GBIF Occurrence Download. https://doi.org/10.15468/dl.gztcbr.

[B3787551] Gertsch W. J. (1941). New American spiders of the family Clubionidae I.. American Museum Novitates.

[B3772545] Watch Global Forest World Resources Institute.. www.globalforestwatch.org.

[B3808401] Gravely F. H. (1924). Some Indian spiders of the family Lycosidae. Records of the Indian Museum.

[B3806490] Guy Y. (1966). Contribution à l'étude des araignées de la famille des Lycosidae et de la sous-famille des Lycosinae avec étude spéciale des espèces du Maroc.

[B3809392] Haddad Charles R., Honiball Allet S., Dippenaar-Schoeman Anna S., Slotow Rob, Van Rensburg Berndt J. (2009). Spiders as potential indicators of elephant-induced habitat changes in endemic sand forest, Maputaland, South Africa. African Journal of Ecology.

[B3785729] Hewitt J. (1916). Descriptions of new South African spiders. Annals of the Transvaal Museum.

[B3805608] Hickman V. V. (1939). Opiliones and Araneae. In: B.A. New Zealand Antarctic Research Expedition 1929-1931. Reports-Series B. Adelaide.

[B4339594] Hoffmann M., Hilton-Taylor C., Angulo A., Böhm M., Brooks T. M., Butchart S. H., Carpenter K. E., Chanson J., Collen B., Cox N. A., Darwall W. R., et al. (2010). The impact of conservation on the status of the world’s vertebrates. Science.

[B4035630] Hoffmann M., Belant J. L., Chanson J. S., Cox N. A., Lamoreux J., Rodrigues A. S. L., Schipper J., Stuart S. N. (2011). The changing fates of the world's mammals. Philosophical Transactions of the Royal Society B: Biological Sciences.

[B3805518] Hogg H. R. (1909). Spiders and Opiliones from the subantarctic islands of New Zealand. The Subantarctic Islands of New Zealand.

[B3788236] Holm A. (1973). On the spiders collected during the Swedish expeditions to Novaya Zemlya and Yenisey in 1875 and 1876.. Zoologica Scripta.

[B3805633] Holm Å. (1962). The spider fauna of the East African mountains. Part I: Fam. Erigonidae. Zoologiska Bidrag från Uppsala.

[B3805742] Holm Å. (1967). Spiders (Araneae) from west Greenland. Meddelelser om Grønland.

[B3788187] Hormiga G. (2000). Higher level phylogenetics of erigonine spiders (Araneae, Linyphiidae, Erigoninae). Smithsonian Contributions to Zoology.

[B4035610] IUCN (2001). IUCN Red List categories v. 3.1.

[B4036139] Subcommittee IUCN Standards and Petitions (2017). Guidelines for using the IUCN RedList categories and criteria.

[B3917214] Jocque R., Dippenaar-Schoeman A. S. (2006). Spider families of the world.

[B3806592] Karsch F (1892). Arachniden von Ceylon und von Minikoy gesammelt von den Herren Doctoren P. und F. Sarasin. Berliner Entomologische Zeitschrift.

[B3804273] Kaston B. J. (1948). Spiders of Connecticut. Bulletin of the State Geological and Natural History Survey of Connecticut.

[B3806723] Keswani S. (2014). Diversity, population and microhabitat used by spiders in citrus agroecosystem. Indian Journal of Arachnology.

[B3806612] Keswani S., Ganesh V. (2014). Diversity, population and habitat used by spiders in banana agro-ecosystems. Indian Journal of Arachnology.

[B3788395] Koch C. L. (1836). Die Arachniden.

[B3788367] Koch L. (1879). Arachniden aus Sibirien und Novaja Semlja, eingesammelt von der schwedischen Expedition im Jahre 1875. Bihang till Kongliga Svenska Vetenskaps-Akademiens Handlingar.

[B4036043] Kuntner M., Rudolf E., Cardoso P. (2017). Nephila antipodiana. The IUCN Red List of Threatened Species.

[B4035459] Lamoreux John, Akcakaya H Resit, Bennun Leon, Collar Nigel J, Boitani Luigi, Brackett David, Braeutigam Amie, Brooks Thomas M, da Fonseca Gustavo A. B, Mittermeier Russell A, Rylands Anthony B, Gaerdenfors Ulf, Hilton-Taylor Craig, Mace Georgina, Stein Bruce A, Stuart Simon (2003). Value of the IUCN Red List. Trends in Ecology & Evolution.

[B4035961] Lewis Owen T., Senior Michael J. M. (2010). Assessing conservation status and trends for the world’s butterflies: the Sampled Red List Index approach. Journal of Insect Conservation.

[B3788276] Locket G. H., Millidge A. F. (1953). British Spiders.

[B3772525] Loksa I. (1965). Araneae. In Ergebnisse der zoologischen Forschungen von Dr. Z. Kaszab in der Mongolei. Reichenbachia.

[B3910646] Lugetti G., Tongiorgi P. (1965). Revisione delle specie italiane dei generi *Arctosa* C. L. Koch e *Tricca* Simon con note su una *Acantholycosa* delle Alpi Giulie (Araneae-Lycosidae). Redia.

[B4035492] Mace Georgina M, Collar Nigel J, Gaston Kevin J, Hilton-Taylor Craig, Akçakaya H Resit, Leader-Williams Nigel, Milner-Gulland E J, Stuart Simon N (2008). Quantification of extinction risk: IUCN's system for classifying threatened species.. Conservation Biology.

[B3917193] Main BY (1985). Further studies on the systematics for Ctenizid trapdoor spiders: a review of the Australian genera (Araneae : Mygalomorphae : Ctenizidae). Australian Journal of Zoology, Supplementary Series.

[B3756962] Malik S, Das S. K., Siliwal M. (2015). Spider (Arachnida: Araneae) fauna of Delhi with first report of cobweb spider *Argyrodes
bonadea* (Karsch, 1881) from India. Indian Journal of Arachnology.

[B3775721] Marples B. J., Marples M. J. (1972). Observations on *Cantuaria
toddi* and other trap-door spiders (Aranea: Mygalomorpha) in Central Otago New Zealand. Journal of the Royal Society of New Zealand.

[B4035558] Martín-López Berta, Montes Carlos, Ramírez Lucía, Benayas Javier (2009). What drives policy decision-making related to species conservation?. Biological Conservation.

[B3775387] Martynovchenko F. A., Mikhailov K. G. (2014). Spiders (Aranei) of Teberda State Reserve: fauna and biotopic distribution.. Euroasian Entomological Journal.

[B3788177] Marusik Y. M., Koponen S., Danilov S. N. (2001). Taxonomic and faunistic notes on linyphiids of Transbaikalia and south Siberia (Araneae, Linyphiidae).. Bulletin of the British Arachnological Society.

[B3788153] Marusik Y. M., Böcher J., Kristensen N. P., Pape T., Vilhelmsen L. (2015). The Greenland Entomofauna. An identification manual of insects, spiders and their allies. Fauna Entomologica Scandinavica..

[B3919681] Marusik Y. M., Böcher J., Kristensen N. P., Pape T., Vilhelmsen L. (2015). Araneae (Spiders) in: The Greenland Entomofauna. An identification manual of insects, spiders and their allies. Fauna Entomologica Scandinavica.

[B3910660] Melic A. (1994). Arañas de Galicia. Boletín SEA.

[B3788285] Miller F. (1947). Pavoučí zvířena hadcových stepí u Mohelna. Archiv Svazu na Výzkum a Ochranu Přírody i Krajiny v Zemi Moravskoslezské.

[B3805653] Miller F. (1970). Spinnenarten der Unterfamilie Micryphantinae und der Familie Theridiidae aus Angola. Publicações Culturais da Companhia de Diamantes de Angola.

[B3788246] Miller F. (1971). Pavouci-Araneida.. Klíč Zvířeny ČSSR.

[B3757209] Miller Jeremy, Griswold Charles, Yin Chang (2009). The symphytognathoid spiders of the Gaoligongshan, Yunnan, China (Araneae: Araneoidea): Systematics and diversity of micro-orbweavers. ZooKeys.

[B3805712] Millidge A. F. (1981). The erigonine spiders of North America. Part 3. The genus *Scotinotylus* Simon (Araneae: Linyphiidae). *Journal of Arachnology*.

[B3806688] More S. B. (2015). Spider diversity of Rundiv, Sidheshwar and Ramnadi area of Chandoli National Park. International Journal of Researches in Biosciences, Agriculture and Technology.

[B3775860] Murphy F., Tongiorgi P. (1979). *Arctosa
villica* (Lucas) 1846: drawings and observations. Bulletin of the British Arachnological Society.

[B3916403] Olson D. M., Dinerstein E., Wikramanayake E. D., Burgess N. D., Powell G. V., Underwood E. C., Loucks C. J. (2001). Terrestrial Ecoregions of the World: A New Map of Life on Earth: A new global map of terrestrial ecoregions provides an innovative tool for conserving biodiversity. BioScience.

[B3805950] Ono H., Matsuda M., Saito H., Ono H. (2009). Linyphiidae, Pimoidae. The Spiders of Japan with Keys to the Families and Genera and Illustrations of the Species.

[B4334526] Padovani L. M. (2010). The Cyclops Islands. Natural Heritage from East to West.

[B3788226] Palmgren P. (1976). Die Spinnenfauna Finnlands und Ostfennoskandiens. VII. Linyphiidae 2. Fauna Fennica.

[B3910800] Pan F., Zheng G., Li S. (2016). Wolf spiders (Araneae: Lycosidae) from Xishuangbanna Tropical Botanical Garden, China. Zoological Systematics.

[B3756952] Patel B. H., Vyas Raju (2001). Spiders of Hingolgadh Nature Education Sanctuary, Gujarat, India. Zoos' Print Journal.

[B3806553] Patel B. H. (2003). Fauna of protected areas of India - I: Spiders of Vansda National Park, Gujarat. Zoos' Print Journal.

[B3806602] Patil S. R., Sambath S., Bhandari R. (2013). Preliminary investigation on spiders (Arachnida: Araneae) in Rani Veerangana Durgawati wildlife sanctuary, Damoh, Madhya Pradesh, India. Indian Forester.

[B4036168] Phillips SJ, Anderson RP, Schapire RE (2006). Maximum entropy modeling of species geographic distributions.. Ecological Modelling.

[B3756722] Platnick N. I., Shadab M. U. (1975). A revision of the spider genus *Gnaphosa* (Araneae, Gnaphosidae) in America.. Bulletin of the American Museum of Natural History.

[B3804036] Platnick N. I., Shadab M. U. (1982). A revision of the American spiders of the genus *Drassyllus* (Araneae, Gnaphosidae). Bulletin of the American Museum of Natural History.

[B3772706] Platnick N. I., Shadab M. U. (1983). A revision of the American spiders of the genus *Zelotes* (Araneae, Gnaphosidae).. Bulletin of the American Museum of Natural History.

[B3756795] Platnick N. I, Murphy J. A. (1984). A revision of the spider genera *Trachyzelotes* and *Urozelotes* (Araneae, Gnaphosidae).. American Museum Novitates.

[B3806583] Pocock R. I. (1900). The fauna of British India, including Ceylon and Burma. Arachnida.

[B3756942] Pocock R. I. (1901). Descriptions of some new species of spiders from British India.. Journal of the Bombay Natural History Society.

[B3775367] Ponomarev A. V., Komarov Y. E. (2013). Preliminary compilation of material on the spider fauna (Aranei) of North Ossetia-Alania. Trudy Severo-Osetinskogo Gosudarstvennogo Prirodnogo Zapovednika.

[B3775357] Ponomarev A. V., Chumachenko Yu. A. (2014). Spiders (Aranei) in hepretobiont mesofauna of the Northwest Caucasus. South of Russia: Ecology, Development.

[B3808512] Qu L. L., Peng X. J., Yin C. M. (2010). One new wolf spiders [sic] of the genus Trochosa (Araneae, Lcosidae) from Yunnan province, China. Acta Zootaxonomica Sinica.

[B3805584] Rainbow W. J. (1917). Arachnida from Macquarie Island. In: Australasian Antarctic expedition 1911-1914. Scientific Reports, Series C.

[B3809421] Rix Michael, Harvey Mark (2010). The spider family Micropholcommatidae (Arachnida: Araneae: Araneoidea): a relimitation and revision at the generic level. ZooKeys.

[B3926248] Rix M. G., Bain K., Main B. Y., Raven R. J., Austin A. D., Cooper S. J.B., Harvey M. S. (2017). Systematics of the spiny trapdoor spiders of the genus *Cataxia* (Mygalomorphae: Idiopidae) from south-western Australia: documenting a threatened fauna in a sky-island landscape. Journal of Arachnology.

[B3804146] Rix M. G., Raven R. J., Main B. Y., Harrison S. E., Austin A. D., Cooper S. J. B., Harvey M. S. (2017). The Australasian spiny trapdoor spiders of the family Idiopidae (Mygalomorphae: Arbanitinae): a relimitation and revision at the generic level. Invertebrate Systematics.

[B3788217] Roberts M. J. (1987). The spiders of Great Britain and Ireland, Volume 2: Linyphiidae and check list.

[B4035481] Rodrigues A., Pilgrim J., Lamoreux J., Hoffmann M., Brooks T. (2006). The value of the IUCN Red List for conservation. Trends in Ecology & Evolution.

[B3788295] Roewer C. F. (1928). Araneae, Echte oder Webespinnen. Die Tierwelt Mitteleuropas.

[B3756930] Roewer C. F. (1959). Araneae Lycosaeformia II (Lycosidae). Exploration du Parc National de l'Upemba, Mission G. F. de Witte.

[B3775771] Roewer C. F. (1960). Araneae Lycosaeformia II (Lycosidae) (Fortsetzung und Schluss). Exploration du Parc National de l'Upemba.

[B3806658] Saha S., Roy T. K., Raychaudhuri D. (2016). Survey on spider faunal diversity of Darjeeling tea plantations. Munis Entomology & Zoology.

[B3805928] Saito H., Ono H. (2001). New genera and species of the spider family Linyphiidae (Arachnida, Araneae) from Japan. Bulletin of the National Science Museum, Tokyo.

[B3775105] Schenkel E. (1963). Ostasiatische Spinnen aus dem Muséum d'Histoire naturelle de Paris. Mémoires du Muséum National d'Histoire Naturelle, Série A, Zoologie (Paris).

[B3777822] Sen S., Dhali D. C., Saha S., Raychaudhuri D. (2015). Spiders (Araneae: Arachnida) of reserve forests of Dooars: Gorumara National Park, Chapramari Wildlife Sanctuary and Mahananda Wildlife Sanctuary. World Scientific News.

[B4412154] Seppälä Sini, Henriques Sérgio, Draney Michael L, Foord Stefan, Gibbons Alastair T, Gomez Luz A, Kariko Sarah, Malumbres-Olarte Jagoba, Milne Marc, Vink Cor J, Cardoso Pedro (2018). Species conservation profiles of a random sample of world spiders I: Agelenidae to Filistatidae.. Biodiversity data journal.

[B3772574] Seyfulina R. R. (2005). A contribution to the knowledge of the spider fauna (Arachnida: Aranei) of Russia: New records for the Amur area. Arthropoda Selecta.

[B3806392] Seyyar O., Oba A., Demir H., Türkeş T. (2016). *Arabelia* Bosselaers, 2009 and *Arabelia
pheidoleicomes* Bosselaers, 2009 (Araneae: Liocranidae) are new records for the Turkish Spider Fauna. Serket.

[B3910820] Siliwal M., Suresh B., Pilo B. (2003). Spiders of Purna Wildlife Sanctuary, Dangs, Gujarat. Zoos' Print Journal.

[B3775751] Simon E. (1876). Les arachnides de France. Paris.

[B3788358] Simon E. (1884). Les arachnides de France.

[B3804067] Simon E. (1895). Arachnides recueillis par M. G. Potanine en Chinie et en Mongolie (1876-1879. Bulletin de l'Académie impériale des sciences de St. Pétersbourg.

[B3808391] Simon E (1905). Arachnides de Java, recueillis par le Prof. K. Kraepelin en 1904.. Mitteilungen aus dem Naturhistorischen Museum in Hamburg.

[B3777812] Song D. X. (1982). Studies on some wolf spiders from China. Sinozoologia.

[B3775135] Song D. X. (1994). New discovery of the male spider of *Drassyllus
excavatus* (Araneae: Gnaphosidae).. Acta Arachnologica Sinica.

[B3777851] Song D. X., Zhu M. S., Chen J. (1999). The Spiders of China.

[B3806902] Srikanth J., Easwaramoorthy S., Kurup N. K., Santhalakshmi G. (1997). Spider Abundance in Sugarcane: Impact of Cultural Practices, Irrigation and Post-Harvest Trash Burning. Biological Agriculture & Horticulture.

[B3757156] Strand E. (1906). Diagnosen nordafrikanischer, hauptsächlich von Carlo Freiherr von Erlanger gesammelter Spinnen. Zoologischer Anzeiger.

[B3757176] Strand E (1908). Nordafrikanische, hauptsächlich von Carlo Freiherr von Erlander gesammelte Lycosiden.. Archiv für Naturgeschichte.

[B3756919] Strand E. (1916). Arachnologica varia, I-IX.. Archiv für Naturgeschichte.

[B3775347] Tanasevitch A. V. (1987). The linyphiid spiders of the Caucasus, USSR (Arachnida: Araneae: Linyphiidae). Senckenbergiana Biologica.

[B3775333] Tanasevitch A. V, Striganova B. R. (1990). The spider family Linyphiidae in the fauna of the Caucasus (Arachnida, Aranei). Fauna nazemnykh bespozvonochnykh Kavkaza. Akaedemia Nauk, pp. 5-114..

[B3772535] Tanasevitch A. V. (2005). New or little-known species of *Agyneta* and *Nipponeta* from Asia (Aranei: Linyphiidae). Arthropoda Selecta.

[B3788167] Tanasevitch A. V. (2008). New records of linyphiid spiders from Russia, with taxonomic and nomenclatural notes (Aranei: Linyphiidae). Arthropoda Selecta.

[B3785743] Tanasevitch A. V. (2013). On linyphiid spiders (Araneae) from Israel. Revue Suisse de Zoologie.

[B3806926] Tanikawa Akio (2006). A new species of the spider genus *Hippasa* (Araneae: Lycosidae) from Yonagunijima Is., the Yaeyama Isls., Japan. Acta Arachnologica.

[B3805973] Thaler K. (1970). Über einige wenig bekannte Zwergspinnen aus den Alpen (Arach., Araneae, Erigonidae). Berichte des Naturwissenschaftlich-Medizinischen Vereins in Innsbruck.

[B3777785] Tikader B. K. (1970). Spider fauna of Sikkim. Records of the Zoological Survey of India 64.

[B3775239] Tikader B. K., Gajbe U. A. (1976). Studies on some spiders of the genus *Zelotes* Gistel from India (family: Gnaphosidae). Proceedings of the Indian Academy of Science.

[B3777423] Tikader B. K., Malhotra M. S. (1980). Lycosidae (Wolf-spiders). Fauna India. (Araneae).

[B3756806] Todd V. (1945). Systematic and biological account of the New Zealand Mygalomorphae (Arachnida). Transactions and Proceedings of the Royal Society of New Zealand.

[B4036158] Tuanmu Mao-Ning, Jetz Walter (2014). A global 1-km consensus land-cover product for biodiversity and ecosystem modelling. Global Ecology and Biogeography.

[B3788266] Tullgren A. (1955). Zur Kenntnis schwedischer Erigoniden.. Arkiv för Zoologi (N.S.).

[B3808491] Turnbull A. L. (1966). A population of spiders and their potential prey in an overgrazed pasture in eastern Ontario. Canadian Journal of Zoology.

[B3804171] IUCN UNEP-WCMC and Protected Planet: The World Database on Protected Areas (WDPA), [On-line], [2017], Cambridge, UK: UNEP-WCMC and IUCN. https://www.protectedplanet.net.

[B4035770] Nations United (2015). The Millenium Development Goals Report.

[B3787581] Agriculture United States Department of Global Desertification Vulnerability Map. https://www.nrcs.usda.gov/wps/portal/nrcs/detail/soils/use/?cid=nrcs142p2_054003.

[B3806645] Vaibhav P. U., Vidyavati M. H., Tanuja K. D., Milind F. N., Karuna G., Veeranagoudar D. K., Pulikeshi M. B. (2017). Spider diversity of Karnatak University campus, Dharwad. Journal of Advanced Scientific Research and Management.

[B3787561] Vetter R. S. (2001). Revision of the spider genus *Neoanagraphis* (Araneae, Liocranidae). Journal of Arachnology.

[B3788386] Walckenaer C. A. (1841). Histoire naturelle des Insects.

[B3808421] WANG DONG, ZHANG ZHI-SHENG (2014). Two new species and a new synonym in the *Pardosa
nebulosa*-group (Lycosidae: Pardosa) from China. Zootaxa.

[B3808471] Whitcomb W. H., Exline H., Ite M. (1963). Comparison of spider populations of ground stratum in Arkansas pasture and adjacent cultivated field. Arkansas Academy of Science Proceedings.

[B3788404] Wider F., Reuss A. (1834). Arachniden. Zoologische miscellen. Museum Senckenbergianum, Abhandlungen aus dem Gebiete der beschreibenden Naturgeschichte.

[B3788256] Wiehle H. (1960). Spinnentiere oder Arachnoidea (Araneae). XI. Micryphantidae-Zwergspinnen. Tierwelt Deutschlands.

[B4036198] Institute World Resources Global Forest Watch. www.globalforestwatch.org.

[B3757242] Catalog World Spider (2017). World Spider Catalog. Natural History Museum Bern.

[B4036024] Catalogue World Spider (2018). World Spider Catalogue. http://wsc.nmbe.ch.

[B3788197] Wunderlich J. (1995). Linyphiidae aus der Mongolei (Arachnida: Araneae).. Beiträge zur Araneologie.

[B3918502] Xu J., Grumbine R. E., Shrestha A., Eriksson M., Yang X., Wang Y. U.N., Wilkes A. (2009). The melting Himalayas: cascading effects of climate change on water, biodiversity, and livelihoods. Conservation Biology.

[B3757197] Yin C. M., Peng X. J., Kim J. P., Wang J. F. (1995). Six new species of the genus *Pardosa* from China (Araneae: Lycosidae). Korean Arachnology.

[B3910810] Yongqiang Z. (1992). Structure and dynamics of spider community in citrus orchard of Nannin. Southwest China Journal of Agricultural Sciences.

